# A Scoping Review of the Relationship Between Psychological (In)flexibility and Living with and Managing Type 1 and Type 2 Diabetes

**DOI:** 10.3390/bs15060792

**Published:** 2025-06-09

**Authors:** Max Z. Roberts, Francesca A. Scheiber, Ashley A. Moskovich, Rhonda M. Merwin

**Affiliations:** Department of Psychiatry and Behavioral Sciences, Duke University School of Medicine, 2400 Pratt St., Durham, NC 27705, USA; max.roberts@duke.edu (M.Z.R.); francesca.scheiber@duke.edu (F.A.S.); ashley.moskovich@duke.edu (A.A.M.)

**Keywords:** psychological flexibility, psychological inflexibility, acceptance and commitment therapy, type 1 diabetes, type 2 diabetes, diabetes distress, diabetes management, glycemic control

## Abstract

Diabetes Mellitus (DM) is highly prevalent and carries a significant self-management burden and elevated risk of biopsychosocial sequelae. Psychological flexibility (PF) has been shown to benefit living with and managing chronic health conditions. The present scoping review aimed to synthesize the available evidence on the relationship between PF and factors central to living with and managing DM. A systematic literature search was conducted. Studies were included if they measured psychological (in)flexibility (PI/PF) and/or one of its component processes and sampled individuals with type 1 or type 2 DM. A total of 48 articles were included. Eighteen (37.5%) sampled individuals with T2D, 16 (33.3%) sampled individuals with T1D, and 14 (29.2%) had mixed diagnostic samples. Twenty-nine (60.4%) reported observational studies, and 19 (39.6%) reported 18 intervention studies. Studies were conducted across 17 countries and broadly found that PI/PF were associated with many clinically meaningful DM variables (e.g., HbA1c, diabetes distress, quality of life, and self-management). Intervention studies including individual, group, and digital Acceptance and Commitment Therapy (ACT) interventions showed trends for beneficial change in PI/PF and diabetes outcomes, but some findings were mixed, and many studies were underpowered. Only two studies tested change in PI/PF as a mediator of diabetes-related outcomes, and most studies used the Acceptance and Action Questionnaire, which has been increasingly criticized for poor discriminant validity. Overall, findings show PI/PF are associated with most aspects of living with and managing diabetes and are generally amenable to change through ACT interventions. However, more methodologically rigorous studies are needed to determine whether PI/PF are active change processes in improving diabetes management and outcomes. Six key calls to action are presented to expand and strengthen this important area of research.

## 1. Introduction

Worldwide, over half a billion people are living with diabetes (IDF Diabetes Atlas, 2025). Diabetes refers to a group of diseases characterized by dysfunction in how the body utilizes glucose. Chronic diabetes conditions include type 1 diabetes (T1D) and type 2 diabetes (T2D), which are both characterized by hyperglycemia, or high levels of glucose in the blood, due to insufficient and/or inefficient insulin production. Although T1D and T2D vary in pathogenesis, they both pose risk for psychosocial sequelae, microvascular complications, macrovascular complications, and premature death if undertreated (Patel, 2024).

Diabetes self-management is an important factor in regulating blood glucose (Davis et al., 2022). For both those with T1D and T2D, self-management involves complex regimens, including checking blood glucose, counting carbohydrates, engaging in physical activity, and taking medication—including insulin for individuals with T1D and insulin-dependent T2D (Davis et al., 2022). These behaviors can help people with diabetes achieve the recommended hemoglobin A1c (HbA1c) level of <7% and prevent or delay medical complications (American Diabetes Association Professional Practice Committee, 2024a). However, many individuals have significant difficulty implementing their regimens (Ahola & Groop, 2013). In the United States, only 50.5% of adults with diabetes have achieved the recommended HbA1c level of <7%, putting the rest at elevated risk for medical complications (American Diabetes Association Professional Practice Committee, 2024a).

Among several barriers to optimal self-management, psychological factors warrant substantial attention (Gonzalez et al., 2016). In a thematic synthesis, individuals with diabetes described self-management as relentless and unpredictable; and they described feeling burdened, panicked, fearful, nervous, helpless, and frustrated (Morales-Brown et al., 2024). These descriptions are unsurprising—the demands of living with and managing diabetes create a uniquely challenging situational context, from which psychological difficulties commonly emerge (Gonzalez et al., 2016).

To elaborate, those with diabetes often receive pressure to maintain euglycemia from medical providers, family members, friends, and themselves (Morales-Brown et al., 2024). However, optimal self-management is behaviorally burdensome and, combined with external pressure, can cause diabetes distress (Gonzalez et al., 2016). For those with T1D, distress can arise from or be amplified by the constant nature of monitoring blood glucose, administering insulin, making decisions about food, eating, and activity, and fearing the possibility of life-threatening hyper- or hypoglycemia (Orben et al., 2022). Persistent hyperglycemia and severe hypoglycemia can lead to both acute (e.g., diabetic ketoacidosis, diabetic coma) and chronic complications (e.g., retinopathy, neuropathy) (American Diabetes Association Professional Practice Committee, 2024b). For those with T2D, distress can arise from or be amplified by difficulties implementing and maintaining significant changes in diet and exercise, self-stigma, and guilt about causing their condition (Orben et al., 2022). Moreover, while self-management behaviors are critical for maintaining euglycemia and staving off medical complications, there are a myriad of factors that can influence blood glucose levels, including several that are beyond control (e.g., sleep quantity and quality, hormonal fluctuations, illness, and even whether one has a sunburn) (American Diabetes Association, 2018). Thus, even when an individual is ‘perfectly’ managing their diabetes, blood glucose levels can be volatile, creating a context of significant unpredictability, especially for those taking exogenous insulin. 

Indeed, up to 42% of individuals with diabetes report elevated diabetes distress (Fisher et al., 2016; Skinner et al., 2020), defined as emotional distress related to living with diabetes, managing laborious regimens, and dealing with uncertainty about long-term complications (Hendrieckx et al., 2021; Polonsky et al., 1995). Importantly, higher levels of distress are associated with reduced levels of self-management (Schmitt et al., 2021); individuals may attempt to cope with distress by avoiding the activities that cue distressing thoughts and feelings (e.g., testing blood glucose levels, counting carbohydrates, and initiating exercise). While this may transiently prevent or reduce distress, avoidance of activities critical to diabetes management inevitably results in elevated blood glucose levels, paradoxically potentiating distress and perpetuating avoidance (Iturralde et al., 2017).

In the current review, we highlight the theoretically central role of psychological flexibility in living well with and adequately managing diabetes and its sequelae. Psychological flexibility refers to the ability to contact the present moment fully and choose and persist in health-promoting, life-enhancing, and values-consistent behaviors, even in the presence of uncomfortable internal and external experiences (Hayes et al., 2006). Inherent in psychological flexibility is a sensitivity to context, or the ability to match actions to constantly changing conditions (Kashdan & Rottenberg, 2010). Given the dynamic needs presented by diabetes, optimal self-care requires a sensitivity to context and flexible responding. This might look like, for example, counting carbohydrates, checking blood glucose, and dosing insulin according to one’s prescribed regimen, even while experiencing diabetes distress, fear of hypoglycemia, and other psychological reactions. 

Psychological inflexibility refers to an unwillingness to experience difficult thoughts and feelings and associated efforts to escape or avoid them and psychological reactions, rather than values, dominating and controlling behavior that produces rigid behavioral responses (Bond et al., 2011; Kashdan & Rottenberg, 2010). For those with diabetes, this might look like managing persistent hyperglycemia by ‘rage bolusing’ or avoiding diabetes distress by forgoing or delaying checking blood glucose, dosing insulin, fulfilling prescriptions, and making dietary changes. Paradoxically, acting to avoid unwanted thoughts and feelings in this way often actually increases distress (Kashdan & Rottenberg, 2010).

Taken together, greater psychological flexibility may help individuals with diabetes open up to the uncomfortable—and normative—distress of living with diabetes, rather than trying to avoid it to detrimental outcome. With greater psychological flexibility, individuals with diabetes may also be better able to adapt behavior to the demands of the situation and choose behaviors that are health-promoting and life-enhancing, rather than those that eventually cause additional suffering (Hadlandsmyth et al., 2013).

Given its clear theoretical role in managing chronic health conditions (see Graham et al., 2016), psychological flexibility—and the therapy designed to target it (Acceptance and Commitment Therapy or ACT; Hayes et al., 2012)—have been studied across a wide range of conditions beyond diabetes, including chronic pain (e.g., McCracken, 2024), obesity (e.g., Lillis & Kendra, 2014), and cancer (e.g., Hulbert-Williams et al., 2015). In general, psychological flexibility has been associated with better coping with health-related distress and health-promoting behavior (Zhang et al., 2018), and changes in psychological flexibility following intervention correspond with improved health outcomes (Gloster et al., 2020; Konstantinou et al., 2023).

In recent years, researchers have increasingly explored the association between psychological flexibility and several diabetes-related outcomes, as well as the efficacy of ACT for improving outcomes in individuals living with diabetes. We undertook this scoping review to answer the following question: What is known about the relationship between psychological flexibility and living with and managing type 1 and type 2 diabetes? The purpose of answering this question is to identify and categorize the available evidence to inform future research and intervention development priorities, ultimately to advance the health and wellbeing of people living with diabetes.

## 2. Materials and Methods

### 2.1. Search and Information Sources

On 28 March 2025, a search following PRISMA-ScR guidelines was conducted by FS in PubMed, PsycINFO, and CINAHL, using multiple iterations of the following search terms: psychological flexibility OR psychological inflexibility OR Acceptance and Commitment Therapy AND diabet* (see Appendix A for full search terms used in each database).

### 2.2. Protocol and Registration

The review protocol was not preregistered.

### 2.3. Eligibility Criteria

Articles were independently screened by two researchers (MR and FS) and were included if they: (1) were original, peer-reviewed, empirical journal articles (i.e., excluding reviews, chapters, protocols, meta-analyses, and grey literature); (2) sampled people with type 1 and/or type 2 diabetes (i.e., excluding studies of parents or caretakers of others with diabetes); (3) quantitatively purported to measure psychological flexibility, or at least one of its component processes, presented within a psychological flexibility theoretical framework; (4) were written in English; and (5) had full text available.

Our inclusion criteria were designed to ensure that we would capture high-quality (peer-reviewed) research directly relevant to the lives of those with type 1 and type 2 diabetes (sample parameters) and would include a measure of psychological flexibility or a component process (measurement methods) to allow for synthesis and evidence integration across the review. Full text and English language criteria were included given practical limitations of accessing and reading the articles. No publication date limit was imposed to maximize the likelihood of identifying a comprehensive, scoping literature base.

### 2.4. Selection of Sources of Evidence

Inclusion decisions were compared following independent screenings. Disagreements on inclusion (almost entirely due to measurement-related questions) were resolved through discussion with a third researcher (AM) until 100% consensus was reached on all articles. Once the list of included articles was finalized, the reference lists of all included articles were screened to identify any additional relevant records. Titles were flagged if they were not previously identified in an earlier stage of the search, were peer reviewed, and appeared relevant (i.e., mentioning that they reported original empirical findings related to diabetes and one of the following: psychological (in)flexibility, or a component process of psychological (in)flexibility, or Acceptance and Commitment Therapy, given psychological flexibility is the target change process of ACT; Hayes et al., 2012). Full texts of these newly identified records were screened as above, followed by a second phase of reference list screening, until no new articles were found. 

### 2.5. Data Items and Charting

After the search process, data extraction proceeded from the articles, including study methodologies and PI/PF measures used, samples and sample sizes, interventions and controls, and main findings. All authors reviewed the data table for emergent patterns and themes in the literature.

### 2.6. Critical Appraisal and Synthesis of Results

Categorization and subsequent synthesis of findings were iterative processes conducted through repeated author discussions and consensus. A formal critical appraisal of each individual article was beyond the scope of this review, but patterns of methodological strengths and shortcomings were sought to evaluate the foundation of current knowledge and inform future research and intervention development priorities.

## 3. Results

### 3.1. Study Selection

**Figure 1:**
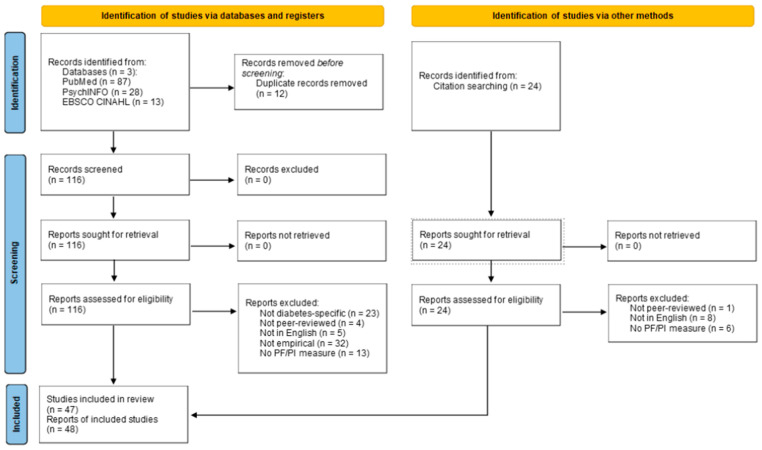
PRISMA-ScR Flowchart.

In total, the initial database searches produced 128 results. After duplicates were removed, 116 articles were retained and screened, of which 39 articles were retained. The reference lists of these 39 articles were screened, identifying 18 new, potentially relevant records. After screening these additional full-text records using the abovementioned criteria, nine were retained. The reference lists of those nine articles were reviewed, leading to another six articles being screened and none of them added. In total, 48 articles were included in the review. Figure 1 details this search and reduction process, including reasons for article exclusions.

### 3.2. Study Characteristics

Of the 48 articles included, 18 (37.5%) sampled individuals with T2D, 16 (33.3%) sampled individuals with T1D, and 14 (29.2%) had mixed diagnostic samples. Most studies (34; 70.8%) sampled middle-aged adults. Twenty-nine (60.4%) reported observational studies, which were all cross-sectional except for one longitudinal study. Nineteen articles (39.6%) reported 18 intervention studies. Of these, seven (36.8%) were single-arm trials, 10 (52.6%) described two-arm trials, one (5.3%) was a three-arm trial, and one (5.3%) was a 2^4^ factorial experiment. Studies were conducted in 17 countries, most frequently in Iran (8; 16.7%), the United Kingdom (7; 14.6%), and the United States (6; 12.5%). Publication dates range from 2007–2025, with half published in the last four years (Median = 2021).

### 3.3. Psychological (In)flexibility Measurement

The most commonly used instrument purported to measure psychological flexibility in this literature base is the Acceptance and Action Diabetes Questionnaire (AADQ; Gregg et al., 2007a), which is a diabetes-specific adaptation of the Acceptance and Action Questionnaire (AAQ; Hayes et al., 2004). In the paper introducing the AADQ, Gregg et al. (2007b) state, “The AADQ measures acceptance of diabetes-related thoughts and feelings and the degree to which they interfere with valued action…” (p. 339). Elsewhere, Gregg et al. (2007b) label the results of the AADQ as “Acceptance, Mindfulness, and Values” (p. 340) and discuss the AADQ results as “diabetes-related acceptance” (p. 340). The subsequent research using the AADQ also varies widely in labeling the construct(s) measured by the AADQ.

While some researchers refer to the AADQ as a measure of psychological flexibility (e.g., Karadere et al., 2019; Merwin et al., 2021), others refer to it as a measure of diabetes acceptance (e.g., Bendig et al., 2021; Saito et al., 2018), diabetes non-acceptance (e.g., Keenan et al., 2022), or both. For instance, Schmitt et al. (2014) stated, “To measure diabetes acceptance, the Acceptance and Action Diabetes Questionnaire, an unevaluated scale assessing non-acceptance, was translated…” (p. 1447). Moreover, various versions and translations of the measure employ a different number of items, and they are scored differently (e.g., reversed or not) and therefore interpreted and described differently. These inconsistencies are also observed across some other PI/PF measures used in these studies (see Table 1).

In addition to or sometimes instead of global measures of psychological flexibility, some studies employed single or multiple measures of psychological (in)flexibility sub-processes. The six sub-processes comprising the psychological flexibility construct include acceptance (willingness to experience all public and private events), present moment awareness (attending fully to the here-and-now), cognitive defusion (allowing thoughts to occur without getting unduly attached to or controlled by them), self-as-context (viewing the self as a changeable, flexible composite transcending unhelpful self-stories), values clarity (knowing and contacting those core principles by which one wants to live), and committed action (choosing and taking steps toward acting in alignment with personal values).

Psychological inflexibility comprises six opposing processes: experiential avoidance (expending effort to escape or avoid unwanted internal experiences even at the expense of valued living), lack of present moment awareness (attending predominantly to the past or future at the expense of contacting the present moment), cognitive fusion (getting unduly attached to or controlled by thoughts in an unworkable way), self-as-content (viewing the self as immutable and narrow and therefore aligning actions with unhelpful self-stories rather than chosen values and environmental contingencies), lack of values clarity (being uncertain about the meaningful principles by which one wants to align their actions), and inaction (behaving under the control of private events or to persistently avoid them at the expense of living in accordance with one’s values).

Some studies specifically measured cognitive fusion or thought believability (Gillanders & Barker, 2019; Gregg et al., 2010; Kioskli et al., 2020; Momeniarbat et al., 2017), diabetes acceptance in isolation of other processes (Schmitt et al., 2018), experiential avoidance in isolation of other processes (Fayyaz & Yusuf, 2023; Gillanders & Barker, 2019; Seo, 2023a; Zandkarimi & Ghahremani, 2022), mindfulness (Alho et al., 2021; Alho et al., 2024; Berlin et al., 2020; Keenan et al., 2022; Kioskli et al., 2020; Momeniarbat et al., 2017; Ryan et al., 2020; Saito & Kumano, 2022), and values clarification, meaning in life, or committed action (Aliche et al., 2023; Gillanders & Barker, 2019; Kioskli et al., 2020; Momeniarbat et al., 2017; Saito & Kumano, 2022; Ryan et al., 2020; Merwin et al., 2021; Styles et al., 2025).

In summary, this body of research is comprised of inconsistencies in the construct description of purported measures of psychological flexibility and intermittent use of measures of component processes, complicating synthesis. To synthesize findings from these studies, we reviewed measure item content and subsequently refer to these measures (listed in Table 1) as measures of psychological flexibility or inflexibility, even if those labels do not align with the label used in the source paper. We also discuss component process findings to the extent available. Measurement issues and opportunities are revisited in the discussion. 

### 3.4. Characterizing Intervention Studies

The review included a total of 18 intervention studies (described in 19 articles) that examined ACT’s effects on PI/PF and component processes and diabetes-related variables (see Table 2). These studies were heterogeneous, with articles reporting results from single-arm trials (n = 7), two-arm RCTs (n = 9), a three-arm RCT (n = 1), and a factorial experiment (n = 1). Details about sample age, diabetes type, treatment modality and dosage, interventionists, intent-to-treat sample sizes, and engagement rates are shown in Table 2. 

In addition to general heterogeneity, some important methodological rigor and feasibility considerations can help contextualize the findings described throughout the remainder of the review. First, intent-to-treat sample sizes of those allocated to an intervention ranged from 6 (Zandkarimi & Ghahremani, 2022) to 57 (Stefanescu et al., 2024a), with a median of 28. The median number of those who completed treatment (excluding two studies for which this information was not reported), which we defined as 100% completion unless otherwise specified by the authors, was 21.5, for a review-wide median completion rate around 76%. Yet, individual study completion rates varied from 16% (Wijk et al., 2023) to 100% (Gregg et al., 2007b; Shayeghian et al., 2016).

High dropout rates (over 50%) before completion were observed in several studies employing self-guided online ACT interventions, even those with psychologist check-in elements (Bendig et al., 2021; Kılıç et al., 2023; Somaini et al., 2023). Other studies with over 50% dropout included a seven-session ACT group intervention that occurred during the COVID-19 pandemic (Wijk et al., 2023) and a 10-session ACT group intervention facilitated by a psychology graduate student (Ryan et al., 2020). The highest treatment completion rates (100%) were in a single, day-long group workshop (Gregg et al., 2007b) and a 10-session ACT + Diabetes Education group (Shayeghian et al., 2016).

The results from these treatment studies must also be considered in light of their methodological rigor, including comparators. None of the RCTs compared ACT-based interventions against another active psychological intervention. Most RCTs had treatment-as-usual (TAU) or waitlist controls, and four compared ACT to diabetes education alone. Yet, even when education was used as an active comparator, the study arms were sometimes unevenly matched in terms of dosage. For example, two studies compared multi-session ACT group interventions against a single diabetes education group session (Ngan et al., 2023; Shayeghian et al., 2016). Although we did not formally grade the quality of evidence in this review, we suggest caution when generalizing broadly beyond the individual study contexts given these methodological shortcomings.

### 3.5. ACT’s Effects on PI/PF

The effects of ACT-based interventions on PI/PF were tested in seven single-arm studies, 10 randomized controlled trials (RCTs) described in 11 articles, and one multicomponent intervention optimization trial. 

#### 3.5.1. ACT’s Effects on PI/PF: Single-Arm Trials

Among the single-arm trials—which were mostly designed to assess feasibility and included small sample sizes—there were significant increases in PF between pre- and post-treatment, with authors reporting medium effect sizes (Ryan et al., 2020; Stefanescu et al., 2024a), large effect sizes (Merwin et al., 2021; Stefanescu et al., 2024a, 2024b), and reliable change (Somaini et al., 2023). As with a change in PF, Somaini et al. (2023) found a reliable reduction in PI. In one study, the difference in pain-specific PF between pre- and post-treatment was not statistically significant in an intent-to-treat sample (Kioskli et al., 2020). 

Four of the single-arm studies reported changes in component processes between pre- and post-treatment, including a significant increase in mindfulness (medium effect size, Ryan et al., 2020); significant increases in valued living (small-to-medium effect sizes, Ryan et al., 2020; Merwin et al., 2021); and a significant decrease in experiential avoidance (Zandkarimi & Ghahremani, 2022). In one study with a high dropout rate, exploratory analyses found statistically significant differences between those who did and did not complete treatment, with treatment completers demonstrating the expected decreases in cognitive fusion and increases in committed action and self-as-context and non-completers demonstrating the opposite pattern (Kioskli et al., 2020). Together, participation in an ACT intervention was consistently associated with improvements in PI/PF or component processes, providing preliminary support for the use of a range of ACT interventions to facilitate change in PF/PI in people living with diabetes. 

#### 3.5.2. ACT’s Effects on PI/PF: Randomized Controlled Trials

Eleven RCTs (described in 12 articles) tested the effects of ACT-based interventions on PI/PF. In seven of eleven trials, ACT-based interventions had a significant effect on PI/PF. These findings showed PF increased with small-to-moderate (Alho et al., 2022) or large effects (Gregg et al., 2007b; Shayeghian et al., 2016; Taheri et al., 2020) but that ACT did not significantly influence mindfulness as a component process (Alho et al., 2022, 2024). In one case, the effect of ACT on PF was delayed, observed at one- and two-year follow-ups but not earlier (Wijk et al., 2023). This study also found significant differences for PF when measured with the AAQ-II but not the AADQ (Wijk et al., 2023). As expected, ACT interventions also significantly decreased PI (Fayazbakhsh & Mansouri, 2019), although in one instance a significant effect became non-significant after controlling for gender (Ngan et al., 2023).

In four of the eleven trials, the effect of ACT on PI/PF was not significant. Two studies were underpowered feasibility trials. In these studies, PF trended toward increasing (Bendig et al., 2021) and PI trended toward decreasing (Kılıç et al., 2023), but neither reached statistical significance. In another study, a three-arm trial comparing a 1-day ACT + diabetes education workshop to education alone or TAU, there was not a significant difference in PI six months post-workshop between TAU and ACT + diabetes education (Whitehead et al., 2017). Finally, in a multicomponent intervention study that involved sessions related to values-guided self-management, the authors reported that there were no significant differences in PF or valued living between those who were allocated to the ACT component and those who were not (Styles et al., 2025). 

To test the theoretical role of PF as an explanatory process in positive diabetes outcomes, two ACT-based intervention studies tested mediation models. Gregg et al. (2007b) found that change in PF significantly mediated the effect of a 1-day ACT workshop on HbA1c. However, Alho et al. (2022) did not find support for process mediation in their study, examining a five-session group ACT intervention. Gregg et al.’s (2007b) post-hoc analyses indicated a significantly greater change in PF in participants who had improved HbA1c versus those who did not.

In summary, RCT findings suggest PI/PF is amenable to change through ACT interventions with people living with diabetes. There is only minimal preliminary evidence supporting its mediating effect for diabetes-related outcomes. Findings sometimes differ based on which measure of PF was used (e.g., Wijk et al., 2023), raising concerns about measurement and construct validity and pointing to an important area for future research. 

### 3.6. Glycemic Control

The association between PF and HbA1c was examined in eight observational studies, and the effect of ACT on HbA1c was investigated in two single-arm trials and five RCTs.

#### 3.6.1. Glycemic Control: Observational Studies

In observational studies, greater PF significantly covaried with lower HbA1c (Alho et al., 2021; Kamody et al., 2018; Keenan et al., 2022; Nicholas et al., 2022; Saito et al., 2018; Saito & Kumano, 2022). In one study, after accounting for age, duration of diabetes, and diabetes self-efficacy, a one standard deviation increase in PF corresponded to a 0.5% decrease in HbA1c for adults with T1D (Nicholas et al., 2022). Similarly, greater PI significantly correlated with higher HbA1c (Keenan et al., 2022; Schmitt et al., 2014, 2018). In one study, the association between PI and HbA1c was significantly stronger than the association between DD and HbA1c (Schmitt et al., 2014). These patterns of associations appeared consistent across diabetes type (e.g., Schmitt et al., 2018).

Cluster and latent profile analyses also suggested that mindfulness and acceptance processes are uniquely important for glycemic control relative to values-based processes (Kamody et al., 2018; Saito & Kumano, 2022). Congruently, after adjusting for diabetes distress, depression, and other demographic and medical covariates (i.e., gender, age, education, diabetes type and duration, and insulin use), diabetes acceptance, measured as a single-component process, accounted for 6.1% of the variance in HbA1c (Schmitt et al., 2018). Importantly, improvements in HbA1c dramatically reduce risk for medical complications, so identifying any related factors, even if they account for a smaller portion of variance, has the potential to create meaningful impact. In sum, in observational studies, PF and PI were consistently associated with HbA1c, and, in some studies, PF and PI accounted for significant variance in HbA1c, even after controlling for other variables known to influence glycemic variation.

#### 3.6.2. Glycemic Control: Single-Arm Trials

Two single-arm treatment studies measured HbA1c pre- and post-treatment (Merwin et al., 2021; Somaini et al., 2023). In their randomized multiple-baseline single case experimental design testing an online ACT intervention, Somaini et al. (2023) recruited participants with T1D and elevated diabetes distress. They reported that, compared to baseline, four out of nine participants achieved a higher percentage of self-monitored blood glucose readings within a specified target range during the intervention phase but not at follow-up. Merwin et al. (2021) tested ACT with T1D patients with eating disorders and reported a trending, but not statistically significant, decrease in HbA1c. They reported clinically significant improvements in HbA1c for individuals above the recommended glycemic levels at baseline. These two single-arm treatment studies had small sample sizes, as they were designed to primarily assess feasibility; however, they provide support for further investigation into the use of ACT to facilitate glycemic control.

#### 3.6.3. Glycemic Control: Randomized Controlled Trials

Five RCTs tested the effects of ACT-based interventions on HbA1c. ACT had a moderate effect on reducing HbA1c in a study examining a five-session group intervention (Alho et al., 2022) and a large effect in a study examining a ten-session group intervention (Shayeghian et al., 2016). Similarly, in a multicomponent intervention study testing the effects of real-time CGM monitoring, sleep extension, healthier snacking support, and values-guided self-management on blood glucose percent time-in-range (TIR), values-guided self-management (i.e., the only ACT intervention component tested) was the only component associated with an increase in TIR of ≥5%. The authors subsequently categorized values-guided self-management as an active intervention component to be included in an optimized self-management intervention (Styles et al., 2025).

In two studies, HbA1c was trending down from pre- to post-intervention, but this decrease did not reach statistical significance (Gregg et al., 2007b; Wijk et al., 2023). However, Gregg et al. (2007b) found that the number of participants with HbA1c < 7% (i.e., in the target range) in the control condition did not significantly change from pre- to post-treatment but significantly increased in the ACT condition. Finally, in a three-arm trial, Whitehead et al. (2017) reported significant between-group differences in HbA1c six months after receiving a 1-day workshop, with the active component groups (i.e., ACT + Education and Education Alone) demonstrating lower HbA1c levels and the TAU group demonstrating higher HbA1c levels. However, post-hoc analyses found that the difference between the ACT + Education condition and the TAU condition was not statistically significant, whereas the difference between the Education Alone condition and the TAU condition was (i.e., only Education Alone significantly reduced HbA1c relative to TAU). Together, these RCT findings provide some preliminary, albeit inconsistent, support for ACT’s effects on HbA1c. 

### 3.7. Diabetes Self-Management

The association between PI/PF and diabetes self-management was tested in nine observational studies, and the effects of ACT on self-management were reported in three single-arm trials and seven RCTs. Diabetes self-management encompasses a range of behaviors, including but not limited to checking blood glucose, taking medications (including insulin), following dietary guidelines, engaging in physical activity, and engaging in foot care. 

#### 3.7.1. Diabetes Self-Management: Observational Studies

Among observational studies, PF was significantly positively associated with self-management behaviors (Saito et al., 2018; Seo, 2023b). Diabetes acceptance, as a component process of PF, was also a significant, unique predictor of self-management behaviors after accounting for diabetes distress, depression, and other covariates; in separate models of self-management behaviors, acceptance accounted for 13.1% to 15.4% of the variation in behaviors (Schmitt et al., 2018). PI was also significantly negatively associated with self-management behaviors (Gillanders & Barker, 2019; Jaworski et al., 2018; Schmitt et al., 2014). Component processes of PI, including fusion (Gregg et al., 2010) and experiential avoidance (Fayyaz & Yusuf, 2023), were also significantly negatively associated with self-management behaviors. However, Saito and Kumano (2022) reported that four clusters representing varying levels of acceptance, mindfulness, and values related to self-management behaviors were not significantly different from each other, indicating some discrepancies in findings. 

In three studies, the authors completed cross-sectional mediation analyses. They found that PI partially mediated the association between blood glucose monitoring and dietary adherence (Jaworski et al., 2018); cognitive fusion partially mediated the effects of self-management on depression symptoms but not vice versa (Gregg et al., 2010); and experiential avoidance fully mediated the association between illness appraisal and self-management (Fayyaz & Yusuf, 2023). Together, PI/PF and their component processes were associated with self-management behaviors across most studies. Moreover, preliminary evidence supports the role of PI/PF and its component processes as mediators of the relationship between self-management behaviors and other diabetes-related variables, although findings are limited due to cross-sectional study designs.

#### 3.7.2. Diabetes Self-Management: Single-Arm Trials

Three single-arm intervention studies measured diabetes self-management pre- and post-treatment (Merwin et al., 2021; Ryan et al., 2020; Somaini et al., 2023). Overall diabetes self-management significantly improved by post-treatment and with a large effect in one study (Merwin et al., 2021); however, another study found only three out of nine participants showed reliable and clinically significant improvement by a one-month follow-up (Somaini et al., 2023). Moreover, physical activity significantly increased, with a large effect in one study (Ryan et al., 2020) and change for five out of nine participants during the intervention phase in another study (Somaini et al., 2023). Time spent sitting also decreased with a moderate effect (Ryan et al., 2020). Together, these single-arm studies provide preliminary support for further investigation into the use of ACT to facilitate improvements in diabetes self-management, a key factor in maintaining glycemic control.

#### 3.7.3. Diabetes Self-Management: Randomized Controlled Trials

Seven RCTs tested the effects of ACT-based interventions on diabetes self-management behaviors. In three studies, ACT had significant, moderate-to-large effects on overall self-management (Gregg et al., 2007b; Shayeghian et al., 2016), as well as physical activity and foot care specifically (Ngan et al., 2023). In a three-arm trial, Whitehead et al. (2017) reported trending, but not statistically significant, between-group differences in self-management. The remaining two-arm trials found no significant effects (Bendig et al., 2021; Kılıç et al., 2023; Wijk et al., 2023), though, as previously noted, two of these studies were low-powered feasibility trials (Bendig et al., 2021; Kılıç et al., 2023). In a multicomponent intervention study, there were no significant differences in self-care behaviors between those who were and were not allocated to the values-guided self-management component (Styles et al., 2025). Thus, some preliminary RCT evidence supports ACT’s effects on improving diabetes self-management, focally including physical activity and foot care.

### 3.8. Diabetes Distress

The association between PI/PF and diabetes distress (DD) was tested in 13 observational studies, and ACT’s effects on DD were tested in two single-arm trials and four RCTs. 

#### 3.8.1. Diabetes Distress: Observational Studies

In observational studies, greater PF was consistently cross-sectionally associated with lower DD (Gillanders & Barker, 2019; Hyesun & Kawoun, 2022; Lee et al., 2021; Nicholas et al., 2022; Saito et al., 2018; Wijk et al., 2024). PF also remained significantly associated with DD after accounting for age, duration of diabetes, and diabetes self-efficacy (Nicholas et al., 2022). Relatedly, greater PI was consistently cross-sectionally associated with higher DD (Aliche & Idemudia, 2024; Karadere et al., 2019; Kılıç et al., 2022; Schmitt et al., 2014). PF also partially, cross-sectionally mediated relations between DD and self-stigma (Hyesun & Kawoun, 2022), as did experiential avoidance as a component process (Seo, 2023a).

A cluster analysis among adults with T1D found that those with a combination of high levels of PF, mindfulness, and values (i.e., high flexibility) had significantly lower DD, with moderate-to-large effects, than (a) those with average levels of PF, mindfulness, and values, (b) those with average levels of PF and mindfulness and low values, and (c) those with average levels of PF and mindfulness and high values. Thus, strengths in all aspects of PF appear to potentially buffer against DD to a greater extent than being high in values clarity alone (Saito & Kumano, 2022). Although the directions of these associations with PI/PF were consistent across diabetes type, one study found that the strength of the relationship between diabetes acceptance and DD was significantly stronger among adults with T1D compared with T2D (Schmitt et al., 2018). 

Despite the consistent patterns of cross-sectional associations described above, Kılıç et al. (2022) found that after controlling for age and baseline DD, baseline PI did not longitudinally predict DD at 6 or 12 months among adults with T2D. These authors posited that the null finding could be due to limited variation in DD across time, leaving little for PI to predict after accounting for baseline DD. In any case, PI’s observational longitudinal prediction of DD has yet to be supported in adults with T2D or tested in adults with T1D. 

#### 3.8.2. Diabetes Distress: Single-Arm Trials

Two single-arm intervention studies measured diabetes distress pre- and post-treatment (Merwin et al., 2021; Ryan et al., 2020). In both studies, diabetes distress had significantly decreased by post-treatment, with a large effect in one study (Merwin et al., 2021) and a small-to-medium effect in another study (Ryan et al., 2020). 

#### 3.8.3. Diabetes Distress: Randomized Controlled Trials

Four RCTs tested the effects of ACT-based interventions on DD. Two studies found that ACT had a moderate effect for decreasing DD (Ngan et al., 2023; Bendig et al., 2021), though one of these studies compared a five-session (10 h total) group ACT + diabetes education intervention to only one session (2 h total) of diabetes education (Ngan et al., 2023) and the other compared a seven-module online intervention + psychologist e-coaching + SMS prompting to waitlist (Bendig et al., 2021). Two studies found no significant effect (Kılıç et al., 2023; Wijk et al., 2023). The results of these latter studies should also be interpreted with caution given low power. In sum, RCT evidence is mixed for the effects of ACT-based interventions on DD.

### 3.9. General Mental Health Symptoms

The association between PI/PF and symptoms of depression, anxiety, stress, and general mental health symptoms was investigated in 15 observational studies, and the effects of ACT on these symptoms were reported in six single-arm intervention studies and five RCTs. 

#### 3.9.1. General Mental Health Symptoms: Observational Studies

In observational studies, greater PF was significantly cross-sectionally associated with lower symptoms of depression (Alho et al., 2021; Imani et al., 2017; Kioskli et al., 2019; Saito et al., 2018), anxiety (Alho et al., 2021; Imani et al., 2017), neuroticism (Momeniarbat et al., 2017), and general psychological distress (Maor et al., 2022; Wijk et al., 2024). Relatedly, greater PI was significantly cross-sectionally associated with higher symptoms of depression (Karadere et al., 2019; Kılıç et al., 2022; Schmitt et al., 2014; Selvarajah et al., 2014), general anxiety (Selvarajah et al., 2014), and death anxiety (Aliche et al., 2023). One study found that greater PI was significantly positively associated with trait but not state anxiety (Karadere et al., 2019).

Component processes of PI/PF consistently followed the directional associations above. For instance, depression symptoms positively correlated with cognitive fusion (Gregg et al., 2010; Kioskli et al., 2019) and negatively correlated with committed action (Kioskli et al., 2019) and diabetes acceptance (Schmitt et al., 2018). The strength of the association between diabetes acceptance and depression did not significantly differ by diabetes type (Schmitt et al., 2018). In a cluster analysis, Saito and Kumano (2022) found that those highest in PF, mindfulness, and values clarity had significantly lower depressive symptoms than those with average levels of those processes or who were high in values clarity alone. However, those with high values clarity combined with average levels of PF and mindfulness had significantly lower depressive symptoms than those with average or lower levels of all processes. Thus, high global flexibility was associated with the lowest depressive symptoms, but high values of clarity alone could confer some benefit as well. 

PI/PF also appear to be incrementally associated with depression after accounting for other relevant variables. For example, in hierarchical regression analyses among adults with painful diabetic neuropathy, the PF step (including measures of PF, fusion, committed action, and self-as-context) significantly accounted for 18.7% of the variance in depression severity and 12.6% of the variance in depression impact after accounting for pain intensity and demographics (Kioskli et al., 2019). Separately, committed action (measured by the activities engagement subscale of the CPAQ) was significantly inversely associated with depression symptoms but not anxiety symptoms after accounting for demographics, pain intensity, QoL, and pain catastrophizing (Selvarajah et al., 2014). 

Moreover, some evidence suggests that PI temporally precedes depression and anxiety in the context of diabetes. In a longitudinal model controlling for age, baseline depression, and self-compassion, Kılıç et al. (2022) found that baseline PI significantly accounted for 10% of the variance in depression symptoms at 6 months but was not a significant predictor of depression symptoms at 12 months. Baseline PI significantly accounted for 6% of the variance in anxiety symptoms at 6 months and 7% of the variance in anxiety symptoms at 12 months. In sum, observational studies found that PI/PF and component processes were consistently associated with depression and anxiety in expected directions. PI has also been shown to longitudinally predict anxiety and, to a relatively shorter duration, depression over time. 

#### 3.9.2. General Mental Health Symptoms: Single-Arm Trials

Six single-arm intervention studies examined depression and anxiety pre- and post-treatment. Most studies found post-treatment improvements; however, most studies were conducted with small samples (i.e., the sample size for five of six studies was ≤30), and all but two focused exclusively on individuals with T1D. Two studies that measured depression found significant improvements in depression at post-treatment, with medium effect sizes (Merwin et al., 2021; Ryan et al., 2020). However, in their study, Kioskli et al. (2020) did not find post-treatment improvements in depression, although exploratory analyses found that treatment completers demonstrated decreases in depression scores.

Similar findings were observed for stress and anxiety. All three studies that measured stress found significant improvements in stress at post-treatment, with a medium effect size (Ryan et al., 2020) and large effect sizes (Stefanescu et al., 2024a, 2024b). While Merwin et al. (2021) did not find significant improvements in anxiety in a sample of adults with T1D and disordered eating, two other studies suggest ACT-based interventions may help improve anxiety in adults with diabetes (small-to-medium effect size; Ryan et al., 2020) and in adults with T1D reporting high levels of diabetes distress, though only 9 participants had data in this small feasibility study (Somaini et al., 2023).

#### 3.9.3. General Mental Health Symptoms: Randomized Controlled Trials

Among the RCTs, two studies found that ACT had moderate-to-large effects reducing generalized anxiety (Alho et al., 2022; Fayazbakhsh & Mansouri, 2019) and a large effect on worry (Fayazbakhsh & Mansouri, 2019). However, in a three-arm trial, between-group differences in anxiety were not statistically different (Whitehead et al., 2017). No support was found for ACT’s effects on reducing symptoms of depression (Alho et al., 2022; Whitehead et al., 2017) or general mental health distress (Wijk et al., 2023). Two underpowered feasibility studies also did not find significant effects for anxiety (Kılıç et al., 2023) or depression (Bendig et al., 2021; Kılıç et al., 2023).

Together, intervention studies provide mixed evidence for the effect of ACT on depression, anxiety, and other mental health symptoms in the context of diabetes. In single-arm trials, receipt of ACT was consistently associated with reductions in depression but not anxiety, whereas in RCTs, receipt of ACT was more consistently associated with reductions in anxiety but not depression.

### 3.10. Quality of Life

The association between PI/PF and quality of life (QoL) was examined in 13 observational studies, and the effects of ACT on QoL in the context of diabetes were tested in one single-arm trial and three RCTs. 

#### 3.10.1. Quality of Life: Observational Studies

Across observational studies, greater PF and its component processes were consistently associated with greater QoL, and greater PI and its component processes were consistently associated with lower QoL. Relationships between PF and QoL did not differ between those with T1D and those with T2D (Schmitt et al., 2014, 2018). More specifically, greater PF was significantly positively associated with several dimensions of QoL, including both mental and physical dimensions (Alho et al., 2021; Berlin et al., 2020; Keenan et al., 2022; Saito et al., 2018; Seo, 2023b). Dimensions of QoL were also positively associated with component processes of PF, including diabetes acceptance (Schmitt et al., 2018), mindfulness (Alho et al., 2021; Berlin et al., 2020; Keenan et al., 2022; Momeniarbat et al., 2017), engaged living (Momeniarbat et al., 2017), and meaning in life (Aliche et al., 2023).

Aligning with these associations, in a sample of youth with T1D, Kamody et al. (2018) used latent profile analysis to derive three distinct profiles related to PF, overall adherence behaviors, and diabetes-specific stress; they found that those high in PF and adherence and low in stress reported higher QoL than both those who were low in PF with moderate adherence and stress and those low in PF and adherence and high in stress. In contrast, Schmitt et al. (2018) reported that diabetes acceptance as a component process was not a concurrent predictor of QoL after accounting for demographic variables, diabetes complications, diabetes distress, and depression. Further, greater levels of PI and cognitive fusion were significantly negatively associated with several dimensions of QoL (Aliche et al., 2023; Aliche & Idemudia, 2024; Berlin et al., 2020; Karadere et al., 2019; Keenan et al., 2022; Kılıç et al., 2022; Momeniarbat et al., 2017; Schmitt et al., 2014).

Of interest, in their longitudinal study with individuals with T2D, Kılıç et al. (2022) reported that PI was a significant, unique predictor of QoL 12 months later, even after accounting for baseline QoL, contributing important information to our understanding of the temporal nature of the relationship between PI and QoL. Finally, three studies found support that PI/PF mediated relations between diabetes-related variables (e.g., glycemic control, diabetes distress, diabetes self-stigma) and QoL (Alho et al., 2021; Aliche & Idemudia, 2024; Seo, 2023b), although the cross-sectional design limits temporal inferences. Together, observational studies find that PI/PF and component processes are consistently associated with QoL in expected directions, and PI may longitudinally predict QoL. 

#### 3.10.2. Quality of Life: Single-Arm Trials

One single-arm study examined change in QoL—Somaini et al. (2023) reported that three out of nine participants demonstrated reliable and clinically significant improvements in QoL one month after intervention; the other six participants demonstrated no change in QoL. 

#### 3.10.3. Quality of Life: Randomized Controlled Trials

Three RCTs investigated the effects of ACT-based interventions on quality of life in the context of diabetes. No studies found significant effects (Alho et al., 2022; Kılıç et al., 2023; Wijk et al., 2023). In sum, at present, very minimal trial evidence in this review shows any effects of ACT on QoL in the context of diabetes.

### 3.11. Diabetes Complications

Four observational studies reported associations between PF and diabetes complications, and one single-arm trial and one RCT examined the effect of ACT on diabetes complications. Diabetes complications included both primary complications (i.e., those directly resulting from diabetes such as chronic fatigue, neuropathic pain, and microvascular/macrovascular complications) and secondary complications (i.e., those deleterious responses to primary diabetes complications that become their own complications, such as pain-related limitations, pain distress, and functional impairment). 

#### 3.11.1. Diabetes Complications: Observational Studies

In their study of adults with T1D and T2D, Schmitt et al. (2018) reported a significant negative association between diabetes acceptance, a component process of PF, and having at least one of the following long-term complications: retinopathy, neuropathy, nephropathy, and/or foot problems. Moreover, those who reported low diabetes acceptance were over twice as likely to report an episode of severe hypoglycemia or hyperglycemia in the past year and at least one long-term complication. In studying chronic fatigue in adults with T2D, Momeniarbat et al. (2017) found that PI and cognitive fusion were significantly positively correlated with fatigue, and mindfulness was significantly negatively correlated with fatigue. Engaged living, however, was not significantly associated with fatigue, suggesting differential influence of component processes. 

In their study of adults with T1D and T2D, Kioskli et al. (2019) examined the association between PF variables—including PF, cognitive fusion, committed action, and self-as-context—and primary and secondary complications—including pain intensity, pain distress, and functional impairment. They found that PF was significantly negatively associated with all three pain variables; committed action was significantly negatively associated with functional impairment; and cognitive fusion was significantly positively associated with pain intensity and functional impairment. Self-as-context was not significantly associated with any of the pain variables. In two separate multiple regression analyses, each accounting for demographic variables and pain intensity, Kioskli et al. (2019) also showed PF incrementally predicted functional impairment but not pain distress. In contrast, in another study of 250 older adults with T1D and T2D, PF and pain-related limitations were not significantly associated with each other (Beiranvand et al., 2023). Overall, these studies suggest that PF and its component processes might play an important role in understanding both primary and secondary diabetes complications.

#### 3.11.2. Diabetes Complications: Single-Arm Trials

In a single-arm treatment study, Kioskli et al. (2020) reported that, in their intent-to-treat analysis, the difference in pain intensity between pre-treatment and post-treatment was not statistically significant. However, exploratory analyses found significant differences between treatment completers and non-completers, with completers demonstrating a decrease and non-completers demonstrating an increase in pain intensity. Kioskli et al. (2020) reported a similar pattern of findings, both for intent-to-treat analysis and exploratory analyses, for pain distress and functional impairment.

#### 3.11.3. Diabetes Complications: Randomized Controlled Trials

One RCT investigated the effects of eight sessions of ACT on PF and pain perception among adults with painful diabetic neuropathy relative to an unspecified control condition. Results showed significant and large effects on reducing pain perception and increasing pain acceptance at post-treatment and follow-up (Taheri et al., 2020).

Together, results from single-arm studies and a randomized controlled trial provide preliminary support for further investigation into the use of ACT to facilitate reductions in primary diabetes complications and reductions in the impact of such complications.

### 3.12. Other Outcomes

In addition to those primary areas of investigation above, single-arm trials and RCTs also measured various other, lesser-investigated outcomes following receipt of an ACT intervention. In single-arm studies, for example, authors reported significant improvements following ACT in eating disorder symptoms (Merwin et al., 2021), positive affect (Ryan et al., 2020), resilience (Ryan et al., 2020), and well-being (Somaini et al., 2023) and significant decreases in alexithymia (Zandkarimi & Ghahremani, 2022). In one study, authors reported a decrease in ‘emotion regulation’ (Zandkarimi & Ghahremani, 2022), but it is not clear to which aspects of emotional regulation they were referring (i.e., based on the questionnaire they utilized, they may have measured change in specific regulation strategies, e.g., rumination, positive reappraisal). One study with children and adolescents found improvements in the patient-doctor relationship (Stefanescu et al., 2024a), but another study with adults did not (Stefanescu et al., 2024b).

In RCTs, support was found for significant ACT-based intervention effects on intolerance of uncertainty (Fayazbakhsh & Mansouri, 2019) but not on fear of hypoglycemia (Wijk et al., 2023), self-efficacy (Ngan et al., 2023), coping strategies (Shayeghian et al., 2016), fear of disease progression (Bendig et al., 2021), understanding diabetes management (Whitehead et al., 2017), or diabetes treatment satisfaction (Whitehead et al., 2017). Lastly, in a multicomponent intervention study, the authors reported that there were no significant differences in snacking or sleep between those who were and were not allocated to the values-guided self-management component (Styles et al., 2025). 

## 4. Discussion

In this review, we presented the available evidence on the relationship between psychological (in)flexibility and diabetes-related variables. This is a robust and growing area of research; half of the 48 studies included were published within the past 4 years. This body of work also represents an international research effort, with studies conducted in 17 different countries. Such sweeping efforts highlight the scope and importance of improving life for the hundreds of millions of people living with diabetes across the globe. 

Findings broadly demonstrate that PF and PI are associated with a range of clinically meaningful, diabetes-related variables. Specifically, correlational findings from 28 studies in this review showed greater PF was consistently associated with lower HbA1c, diabetes distress, depression, anxiety, and diabetes complications; it was also consistently associated with a higher quality of life, along with more engagement in self-management behaviors and adaptive coping. Psychological *in*flexibility consistently showed these same associations in the opposite direction. There was one observational longitudinal study included, which provided initial support for PI as a temporal predictor of anxiety, depression, and quality of life in the context of diabetes. Further, findings preliminarily support that PI/PF and various diabetes-related outcomes are amenable to change through ACT-based interventions.

Nineteen articles described 18 intervention studies of the effects of ACT-based interventions on PI/PF or their component processes and a wide range of diabetes-related variables. Preliminary support (including non-significant signals or trends) was found for ACT improving HbA1c, diabetes distress, depression and anxiety, self-management, and living with diabetes complications, including neuropathic pain. Contrasting with prior research (e.g., Konstantinou et al., 2023), minimal evidence supported the effect of ACT on QoL in the context of diabetes, highlighting a critical area of future research. Lastly, only two intervention studies tested PF as a mediator of the effects of ACT on HbA1c, a key diabetes outcome, and there were differences in the outcomes of these studies. Specifically, change in PF explained reductions in HbA1c in one study (Gregg et al., 2007b) but not another (Alho et al., 2022). In total, ACT appears to have some benefits for individuals living with diabetes, although findings are not consistent enough or of high enough quality across studies in any one area to declare definitive efficacy. 

These interventions further produced a variety of discrepant findings, including around ACT’s expected effects on PI/PF, through which diabetes-related variables are theoretically influenced. For example, there were no effects of ACT observed on PI/PF among online studies with high dropout rates (Bendig et al., 2021; Kioskli et al., 2020), and one study found that the effect on PI became non-significant after controlling for gender (Ngan et al., 2023), warranting additional inquiry. Following a 1-day ACT + Diabetes Education workshop, Gregg et al. (2007b) also found a large effect on PF at 3 months, while Whitehead et al. (2017) found a trending but not statistically significant effect on PF at 6 months. Better understanding the factors involved in different process outcomes such as these could improve our ability to tailor and/or maximize intervention effectiveness.

Elsewhere, at least one study found that the effect of an ACT-based intervention on PI/PF was not observable until the 1- and 2-year follow-up (Wijk et al., 2023). Such delayed effects have been observed in other ACT intervention studies, suggesting that in some cases, it might take time for PI/PF to integrate (Gloster et al., 2020). Thus, the sampling timeframe could be another factor differentiating outcomes. In sum, while intervention studies provide preliminary support for ACT’s effects on PI/PF and important diabetes outcomes, evidence is occasionally mixed, and minimal evidence supports process mediation of ACT’s effects on diabetes outcomes through moving PI/PF. Further examining discrepant outcomes and how these studies differ (in samples, intervention, interventionist, or other characteristics) could improve our understanding of when and how PI/PF relate to diabetes outcomes and our subsequent ability to effect positive change.

This literature base has several consistent shortcomings that could provide additional context for these findings. Intervention studies are variable in terms of methodological design rigor, power, modality, and dosage, with online intervention modalities having particularly high dropout rates. Thus, while the findings often appear promising and align with conceptualizing PI/PF as a relevant factor in the context of chronic health conditions (e.g., Kashdan & Rottenberg, 2010; Hulbert-Williams et al., 2015), they are inconsistent. This takeaway aligns with other ACT/PF for chronic disease reviews (e.g., Graham et al., 2016) and highlights a need for high-powered studies with more rigorous methodologies (see Hayes et al., 2021 for guidance). For instance, even significant findings in RCTs are sometimes limited by dosing imbalances between intervention and control conditions. That is, studies that tested ACT-based interventions against diabetes education as an active control sometimes compared multiple sessions of ACT to single sessions of diabetes education (Ngan et al., 2023; Shayeghian et al., 2016), limiting enthusiasm for the findings. 

Another major limitation is that most studies used a variant of the Acceptance and Action Questionnaire (AAQ; Hayes et al., 2004) as the primary measure of PI/PF (e.g., AADQ, AAQ-II). Although problem-specific variants of the AAQ-II (such as the AADQ for diabetes) have better psychometric properties (Ong et al., 2019), the AAQ and its variants are widely criticized for their poor construct and discriminant validity (e.g., Cherry et al., 2021; Tyndall et al., 2019; Wolgast, 2014). One study in this review, for example, found significant differences between ACT intervention and a control condition on the AAQ-II but not the AADQ (Wijk et al., 2023). Beyond psychometric criticism of these measures, the present review found that the number of items and precise descriptions of what was being measured by the same scale changed from study to study, limiting the construction of a cumulative and trustworthy knowledge base (Cherry et al., 2021).

Before turning to future directions, the limitations of the review itself must be considered. First, the heterogeneity of samples, measures, problems, and methodologies limited our ability to synthesize findings into a coherent whole and present objective recommendations for future directions. Yet, we believe the present review highlights the wide scope and status of this diverse literature. Second, we included only studies that measured PI/PF, omitting others that report the effects of ACT on diabetes outcomes but did not include a process measure (e.g., Maghsoudi et al., 2019). Third, and relatedly, whether a measure actually measures PI/PF was discussed at length within our team, reflecting field-wide discussions on what purported PI/PF measures measure (Cherry et al., 2021). The findings of this review, and indeed the literature base, depend on measurement, and this should be considered a central issue. Fourth, we presented studies equivalently within the review, without any form of weighting, despite significant differences in quality, though we attempted to caution or caveat findings where applicable when methodological rigor was questionable. Some studies, however, were missing information that enabled total determination of their rigor but were presented within the review to obtain a scoping base.

Considering the limitations of the review and literature base alongside the more promising findings, notable areas of opportunity abound. To fulfill the goal of this review, we deduced the following recommendations/calls to action for future research and intervention development emerging from the review findings: 

**Measurement**: When measuring PI/PF in intervention studies, researchers are encouraged to select a multidimensional measure with adequate psychometric properties (e.g., MPFI; CompACT; PPFI). To reiterate a growing consensus in the field, the AAQ and its variants should no longer be used as a measure of PI/PF (Cherry et al., 2021), although the debate continues (Ruiz et al., 2024).

**Analyses**: When analyzing intervention study results, researchers are encouraged to report process mediation models, especially including multidimensional measures of psychological (in)flexibility. Some findings in this review suggest specific components of psychological flexibility, such as values clarity, could be particularly useful in diabetes-related management (e.g., Styles et al., 2025); multiple mediation models would enable more robust and actionable conclusions. Given the high dropout rate observed in some studies, we also encourage application of intent-to-treat analyses to minimize bias. 

**Comparators**: When testing the effects of an ACT-based intervention against a comparator in an RCT, researchers are encouraged to at least include dose-matched diabetes education as an active control rather than a waitlist or TAU. Whitehead et al. (2017) found that education alone compared with TAU significantly reduced HbA1c. Thus, studies showing superiority of ACT outcomes relative to diabetes education are more promising than those showing superiority to waitlist or TAU. However, whether ACT’s effects exceed other plausibly beneficial psychological interventions has yet to be determined. An important future step will be conducting non-inferiority trials comparing ACT to other active psychological interventions in the context of diabetes. Other, more idiographic approaches would also contribute meaningfully to advancing our understanding of ACT’s effects on diabetes-related outcomes. These include multiple baseline designs (e.g., Somaini et al., 2023), multicomponent intervention trials (e.g., Styles et al., 2025), or those employing high-density longitudinal design and experience sampling methods. 

**Engagement**: Given the low treatment engagement observed in some intervention studies, particularly those of online interventions, researchers are encouraged to incorporate more provider-client touchpoints and supplemental communication to improve online intervention adherence. In addition, or as an alternative, reducing the number of sessions is likely to confer benefits. The highest treatment completion rate, 100%, was found in a single-session group intervention (Gregg et al., 2007b), though another single-session group intervention found only 72% of those allocated to the intervention engaged in it (e.g., Whitehead et al., 2017). Thus, additional work is needed to better understand and improve factors related to treatment engagement.

**Expansion**: A major benefit of ACT relative to other psychosocial intervention systems is its transdiagnostic, broad-spectrum applicability. Yet, many studies excluded participants with other serious medical comorbidities, limiting effectiveness determinations and generalizability to real-world settings where such comorbidities with diabetes are common. Thus, we encourage less stringent exclusion criteria in ACT intervention studies. Similarly, high-impact and common comorbidities with diabetes, such as eating disorders, are under-researched and present important areas for future work. 

**Tailoring**: The high heterogeneity of ACT-based interventions described in this review presents a challenge for synthesis but a potential benefit for implementation. For instance, shifting modality and even credentials of the facilitator to match the setting implementation needs and population preferences is an advantage for treatment uptake in real-world settings. We therefore encourage continuing to test creative, tailored intervention structures, ensuring that other recommendations above are followed to aid in robust findings and evidence synthesis (see Hayes et al., 2021, for additional methodological recommendations). 

Looking to the future, if the evidence continues to evolve to suggest that increased psychological flexibility contributes to living well with diabetes, more research could be allocated to translational clinical applications. This might include, for example, better assessment of psychological flexibility in persons living with diabetes with elevated glycemic levels and associated in-clinic interventions. Assessment and intervention in real-world settings where diabetes-related psychosocial sequelae are most likely encountered (e.g., primary care, endocrinology) could have a significant impact on health outcomes at scale. Innovations might include brief, even single-session interventions or digitalized interventions increasing psychological flexibility in the context of diabetes management. Such interventions should be made widely accessible to match the scope and scale of the challenges of living with diabetes.

## 5. Conclusions

The present review found robust evidence for the consistent association of psychological (in)flexibility and a range of important diabetes-related variables. Evidence for the effects of ACT-based interventions on diabetes outcomes is promising but preliminary given methodological shortcomings in many intervention studies. Recommendations for future research and intervention development were presented to shore up the research in this important area and to continue moving toward a science and practice that improves the lives of the hundreds of millions of people living with and managing type 1 and type 2 diabetes. 

## Figures and Tables

**Table 1:** Global Psychological (In)Flexibility Measures Used in Diabetes Research *.

	Primary Construct Label(s) in Source Text	Reference	Scale Language	# of Items
**Acceptance and Action Diabetes Questionnaire (AADQ)**				
	Acceptance	Bendig et al. (2021)	N/A	11
	Acceptance, Mindfulness, and Values	Gregg et al. (2007b)	English	11
	Acceptance	Jaworski et al. (2018)	Polish	N/A
	Acceptance	Kamody et al. (2018)	English	6
	Psychological Flexibility and Acceptance/Psychological Inflexibility and Experiential Avoidance	Karadere et al. (2019)	Turkish	9
	Non-Acceptance	Keenan et al. (2022)	English	9
	Psychological Flexibility	Merwin et al. (2021)	English	11
	Acceptance	Saito & Kumano (2022)	Japanese	8
	Acceptance	Saito et al. (2018)	Japanese	8
	Acceptance/Non-Acceptance	Schmitt et al. (2014)	German	6
	Acceptance/Non-Acceptance	Schmitt et al. (2018)	German	6
	Acceptance	Shayeghian et al. (2016)	N/A	11
	Acceptance	Stefanescu et al. (2024a)	Romanian	6
	Acceptance	Stefanescu et al. (2024b)	Romanian	6
	Acceptance/Non-Acceptance	Whitehead et al. (2017)	English	11
	Psychological Flexibility	Wijk et al. (2023)	Swedish	11
	Psychological Flexibility and Acceptance	Wijk et al. (2024)	Swedish	6
**Acceptance and Action Diabetes Questionnaire—Youth Self-Report (AADQ-YR)**				
	Psychological Flexibility/Acceptance and Non-acceptance	Berlin et al. (2020)	English	9
	Psychological Flexibility/Diabetes Rejection and Acceptance	Rajaeiramsheh et al. (2021)	Persian	9
**Acceptance and Action Diabetes Questionnaire—Parent Report (AADQ-PR)**				
	Psychological Flexibility/Acceptance and Non-acceptance	Berlin et al. (2020)	English	9
	Psychological Flexibility/Diabetes Rejection and Acceptance	Rajaeiramsheh et al. (2021)	Persian	9
**Acceptance and Action Questionnaire (AAQ)**				
	Acceptance Action	Hyesun & Kawoun (2022)	Korean	16
	Acceptance and Action	Lee et al. (2021)	Korean	16
	Acceptance Action	Seo (2023b)	Korean	16
**Acceptance and Action Questionnaire 2 (AAQ-II)**				
	Experiential Avoidance	Aliche and Idemudia (2024)	English	7
	Experiential Avoidance	Aliche et al. (2023)	N/A	7
	Experiential Avoidance	Fayazbakhsh & Mansouri (2019)	N/A	7
	Variety, Acceptance, Experiential Avoidance, Psychological Flexibility, No Experiential Avoidance, Acceptance and Action	Imani et al. (2017)	N/A	N/A
	Psychological Inflexibility	Kılıç et al. (2022)	English	7
	Psychological Inflexibility	Kılıç et al. (2023)	English	7
	Psychological Flexibility	Momeniarbat et al. (2017)	Persian	7
	Psychological Inflexibility	Ngan et al. (2023)	Chinese	7
	Psychological Inflexibility and Experiential Avoidance	Nicholas et al. (2022)	N/A	7
	Psychological Flexibility	Ryan et al. (2020)	English	7
	Psychological Flexibility	Wijk et al. (2023)	Swedish	6
	Psychological Flexibility	Wijk et al. (2024)	Swedish	6
**Acceptance and Action Questionnaire—Stigma (AAQ-S)**				
	Psychological Flexibility and Inflexibility	Lee et al. (2021)	Korean	21
**Avoidance and Fusion Questionnaire—Youth (AFQ-Y)**				
	Psychological Inflexibility	Berlin et al. (2020)	English	17
	Avoidance Behaviors and Fusion	Keenan et al. (2022)	English	17
	Psychological Inflexibility	Rajaeiramsheh et al. (2021)	Persian	N/A
**Chronic Pain Acceptance Questionnaire (CPAQ-7)**				
	Acceptance ^1^	Beiranvand et al. (2023)	N/A	7
**Chronic Pain Acceptance Questionnaire 8 (CPAQ-8)**				
	Acceptance	Kioskli et al. (2020)	English	8
	Acceptance	Kioskli et al. (2019)	English	8
**Chronic Pain Acceptance Questionnaire (CPAQ)**				
	Ability to Acknowledge and Adapt to Chronic Pain	Selvarajah et al. (2014)	English	20
	Acceptance	Taheri et al. (2020)	Persian	N/A
**Children’s Psychological Flexibility Questionnaire (CPFQ)**				
	Psychological Flexibility	Stefanescu et al. (2024a)	Romanian	24
**Diabetes Acceptance and Action Scale (DAAS)**				
	Psychological Flexibility	Alho et al. (2021)	Finnish	42
	Psychological Flexibility	Alho et al. (2022)	Finnish	42
	Psychological Flexibility	Alho et al. (2024)	Finnish	42
	Psychological Flexibility	Berlin et al. (2020)	English	42
	Psychological Flexibility/Acceptance	Gillanders & Barker (2019)	English	42
**Diabetes Acceptance and Action Scale 22 (DAAS-22)**				
	Psychological Flexibility	Berlin et al. (2020)	English	22
	Psychological Flexibility	Keenan et al. (2022)	English	22
	Acceptance and Action	Rajaeiramsheh et al. (2021)	Persian	22
**Diabetes Acceptance and Action Scale Revised (DAAS-R)**				
	Psychological Flexibility/Acceptance	Gillanders & Barker (2019)	English	9
	Acceptance	Styles et al. (2025)	N/A	9
**Diabetes Acceptance and Action Scale 9 (DAASito-9)**				
	Psychological Flexibility	Keenan et al. (2022)	English	9
**Diabetes Acceptance and Action Scale 3 (DAASito-3)**				
	Psychological Flexibility	Keenan et al. (2022)	English	3
**Multidimensional Psychological Flexibility Inventory (MPFI)**				
	Psychological Flexibility and Inflexibility	Somaini et al. (2023)	English	60
	Psychological Flexibility and Inflexibility	Stefanescu et al. (2024b)	Romanian	60
**Psychological Flexibility Questionnaire (PFQ)**				
	Psychological Flexibility	Maor et al. (2022)	N/A	20

*Note*. ^1^ These authors describe using a version of the CPAQ adapted for use in elderly populations (adapted by Shirazi et al. (2015) as cited in Beiranvand et al. (2023)), though we were unable to retrieve that source article in English to confirm its origins and relationship with the original CPAQ. N/A = This information was not readily available within the manuscript text. # of Items = the number of items included in each version of each questionnaire. * The same article may be referenced multiple times in the table, corresponding to the number of distinct PI/PF measures included in the study.

**Table 2:** ACT-Based Intervention Studies for Diabetes-Related Outcomes.

Authors (Year)	Treatment	Sample	Tx Modality/Format	Facilitator(s)	# Sessions	Length of Session(s)	# Intent to Treat	# Labeled Completers	# Analyzed	Control	Main Findings
**Single-Arm Trials**											
Kioskli et al. (2020)	ACT	T1D/T2D + PDN Adults	Online + 1:1 Teletherapy	Self-Guided + Master’s therapist	8 modules + 2 sessions	12–35 min. modules + 30–45 min. sessions	30	12	30		In an ITT analysis, changes in pain-specific PF, pain distress, depression, pain intensity, and functional impairment were not significant. However, changes in cognitive fusion, committed action, and self-as-context were significant, with participants demonstrating higher levels of fusion and lower levels of committed action and self-as-context; deterioration was largely attributed to those who did not complete treatment. Posthoc analyses showed that the interaction between time point and treatment completion was significant for all variables, except pain acceptance, with completers demonstrating expected improvements. Treatment completers reported improvements, while treatment non-completers reported no change in health and functioning.
Merwin et al. (2021)	ACT	T1D ED + Adults	Mobile app + 1:1 F2F	Self-Guided + Psychologist	12 + 3 optional sessions + app use between sessions	50–60 min	28	20	19		Participants demonstrated significant increases in PF, values progress, and diabetes self-management and significant decreases in values obstruction, diabetes distress, and depression. Changes in HbA1c and anxiety were not significant.
Ryan et al. (2020) ^1^	ACT	T1D/T2D Adults	F2F Group	Graduate Psychology Student	10 sessions	60–120 min	28	11	20		Participants demonstrated significant increases in PF, valued living, mindfulness, and physical activity. They demonstrated significant decreases in diabetes distress, sitting time, depression, anxiety, and stress. Participants reported high levels of satisfaction with the intervention.
Somaini et al. (2023)	ACT	T1D Adults	Online + Email + Phone	Self-guided + Psychologist	6 modules + 3 check-in calls	Module time N/A, 5–15 min. call time	25	9	9	MBD	Of nine participants, PF significantly increased for 7 participants, and there was a reliable decrease in PI. There was reliable change in diabetes self-management for 3 participants and QoL for 3 participants. Participants rated the intervention as highly acceptable.
Stefanescu et al. (2024b)	ACT	T1D Adults	1:1, Zoom	Psychologist	4 sessions	45 min	47	37	13		Participants demonstrated significant increases in PF and significant decreases in PI and diabetes-associated stress. Participants reported being satisfied with the intervention.
Stefanescu et al. (2024a)	ACT	T1D Children and Adolescents	1:1, F2F or Teletherapy Mixed	Psychologist	4 sessions	50 min	57	55	55		Participants demonstrated significant increases in PF and significant decreases in PI and stress. Participants reported that the intervention was helpful.
Zandkarimi and Ghahremani (2022)	ACT	T1D Adults	1:1, F2F	N/A	8 sessions	60 min	6	N/A	N/A		Participants demonstrated a significant decrease in experiential avoidance and significant improvement in glycemic control.
**Randomized Controlled Trials (RCTs)**											
Alho et al. (2022, 2024)	ACT	T1D Adolescents	F2F Group	Psychologist	5	90 min.	36	28	31	TAU	Changes in PF, glycemic control, and anxiety were significantly different between the ACT + TAU group and the TAU group, favoring the ACT + TAU group. Within the ACT group, PF increased, and HbA1c and anxiety decreased. Changes in acceptance and mindfulness skills, depression, and QoL were not significantly different between groups. Change in PF did not mediate the effect of the intervention on glycemic control or anxiety. Participants reported being highly satisfied with the intervention.
Bendig et al. (2021)	ACT	T1D/T2D Adults	Online + e-coach + SMS	Self-Guided + Psychologist	7 modules	45–60 min.	21	9	12	WL	Changes in diabetes distress, but not PF, diabetes self-management, depression, anxiety, or QoL, were significantly different between the ACT group and the waitlist control group, favoring the ACT group for diabetes distress. Participants reported that their well-being improved as a result of the intervention.
Fayazbakhsh and Mansouri (2019)	ACT	T2D Adults	N/A	N/A	8 sessions	90 min.	24	N/A	N/A	“Not in any treatment”	Changes in experiential avoidance, anxiety, and worry were significantly different between groups, favoring the ACT group. Those in the ACT group demonstrated reductions in these variables.
Gregg et al. (2007b)	ACT + Ed.	T2D Adults	F2F Group	Psychologist	1	7 h.	43	43	40 (HbA1c) 36 (measures)	Ed.	Changes in PF, HbA1c <7%, and diabetes self-management, were significantly different between the ACT + Education group and the Education Alone group, favoring the ACT group. Within the ACT group, PF and self-management increased. Moreover, the proportion of participants with HbA1c <7% increased in the ACT group. Change in HbA1c was mediated by changes in diabetes self-management and PF. Participants reported being satisfied with both conditions.
Kılıç et al. (2023)	ACT + SC	T2D Adults	Online + Optional HW booklet	Self-Guided	5	30 min. + 10–15 min. optional practice	19	6	6 (5-week follow-up) 4 (9-week follow-up) 2 (optional interview)	WL	Participants in the ACT group demonstrated moderate improvements in diabetes distress and small improvements in PI, depression and anxiety. Participants in the waitlist control group demonstrated small improvements in diabetes distress and PI. Due to difficulties with treatment completion and trial retention, the authors note that treatment effects are difficult to interpret.
Ngan et al. (2023)	ACT + Ed.	T2D Adults	F2F Group	N/A	5 sessions	120 min.	24	21	24	Ed.	Changes in PI, diabetes distress, and diabetes self-management were significantly different between the ACT + Education group and the Education Alone group, favoring the ACT + Education group. The effect of ACT on PI became non-significant after controlling for gender. Participants reported that they felt the intervention improved their psychological well-being and understanding of diabetes.
Shayeghian et al. (2016)	ACT + Ed.	T2D Adults	F2F Group	Psychologist	10	120 min.	53	53	50	Ed. + TAU	Participants in the ACT + Education group demonstrated significantly higher PF and diabetes self-management and significantly lower HbA1c than those in the Education + TAU group. There was a significant interaction effect, such that only those with an effective coping style improved their self-management after participating in the intervention.
Taheri et al. (2020)	ACT	Unspecified Type, PDN Adults	N/A	Psychologist	8 sessions	N/A	25	22	20	N/A	Changes in PF and pain perception were significantly different between groups, favoring the ACT group. Within the ACT group, pain acceptance increased, and pain perception decreased.
Wijk et al. (2023)	ACT	T1D Adults	F2F Group + Homework	Psychologist + Diabetes Nurse	7 sessions	120 min.	43	7	43	TAU	The interaction between time and group affiliation was significant for PF on the AAQ-II, favoring the ACT group, but not on the AADQ. The interaction between time and group affiliation was not significant for HbA1c, diabetes distress, diabetes self-management, or QoL.
Whitehead et al. (2017)	ACT + Ed.	T2D Adults	F2F Group	Mental health nurse	1 workshop	6.5 h.	54	39	36 (HbA1c) 39 (measures)	Ed. + TAU, and TAU	There were no significant differences between the three conditions in PF, depression, or anxiety. Participants in the ACT + Education group and the Education Alone group demonstrated reductions in HbA1c, but only the difference between the Education Alone group and the control group was significant. There was a trending, but not significant, difference between the ACT + Education group and the Education group, favoring the ACT + Education group.
Styles et al. (2025) ^a^	Values, CGM, Sleep, Snacking	T1D Adolescents + Young Adults	1:1, F2F or Teletherapy	“Trained Staff”	3 sessions	N/A	28	24	21	Factor Controls	The values-guided intervention component was the only component tested that was associated with an increase in time-in-range for blood glucose. Differences in PF and diabetes self-management, between those who received the values component and those who did not, were not significant.

*Note*: Completers were defined as those who completed between 80–100% of the intended intervention. F2F = face-to-face or in-person. TAU = treatment as usual. WL = waitlist. Ed. = Diabetes education. SC = self-compassion. N/A = this information was not readily available in the text. Grey table cells indicate that the information was not applicable due to study design. MBD = multiple baseline design. # Sessions = the number of sessions or modules in the intervention. ^a^ Note: this study was a multicomponent intervention trial, with a 2^4^ factorial design. ^1^ Ryan et al. (2020) used a threshold of *p* < 0.01 for statistical significance. For ease of synthesis across studies, we used *p* < 0.05 to indicate statistical significance. Our presentation of the Ryan et al. (2020) study should be interpreted with this in mind.

## Data Availability

No new data were generated during this study. Additional data organization materials available upon request.

## Appendix A

PubMed Search Terms

(“Acceptance and Commitment Therapy”[MeSH Terms] OR “acceptance and commitment therapy”[All Fields] OR “acceptance and commitment therapy*”[Title/Abstract] OR “psychological flexibility”[All Fields] OR “psychological inflexibility”[All Fields] OR “psychological flexibility*”[Title/Abstract] OR “psychological inflexibility*”[Title/Abstract]) AND (“Diabetes Mellitus”[MeSH Terms] OR “Diabetes”[All Fields] OR “Diabetes Insipidus”[MeSH Terms] OR “Diabetes Complications”[MeSH Terms] OR “diabetes mellitus, type 2”[MeSH Terms] OR “diabetes mellitus, type 1”[MeSH Terms] OR “Latent Autoimmune Diabetes in Adults”[MeSH Terms] OR “Diabulimia”[MeSH Terms] OR “diabet*”[Title/Abstract])

PsycINFO Search Terms

(DE “Acceptance and Commitment Therapy” OR DE “Psychological Flexibility”) AND (DE “Diabetes” OR DE “Type 1 Diabetes” OR DE “Type 2 Diabetes” OR DE “Diabetes Mellitus” OR DE “Diabetes Insipidus”)

CINAHL Search Terms

(DE “Acceptance and Commitment Therapy” OR DE “Psychological Flexibility”) AND (DE “Diabetes” OR DE “Type 1 Diabetes” OR DE “Type 2 Diabetes” OR DE “Diabetes Mellitus” OR DE “Diabetes Insipidus”)
